# Reduction in postoperative complications by robotic surgery: a case–control study of robotic versus conventional laparoscopic surgery for gastric cancer

**DOI:** 10.1007/s00464-021-08483-1

**Published:** 2021-04-12

**Authors:** Takahiro Kinoshita, Reo Sato, Eigo Akimoto, Yuya Tanaka, Takafumi Okayama, Takumi Habu

**Affiliations:** Gastric Surgery Division, National Cancer Center Hospital East, 6-5-1 Kashiwanoha, Kashiwa, 277-8577 Japan

**Keywords:** Gastric cancer, Robotic surgery, Laparoscopic surgery, Postoperative complication

## Abstract

**Background:**

Robotic gastrectomy (RG) is being increasingly performed globally; it is considered an evolved type of conventional laparoscopic surgery with excellent dexterity and precision, but higher costs and longer operation time. Thus, there is a need to identify the benefits from RG and its specific candidates.

**Methods:**

This retrospective study analyzed data from a prospectively collected clinical database at our center. Data of patients with primary gastric cancer undergoing either robotic or laparoscopic radical gastrectomy from June 2014 to June 2020 were reviewed. Surgical outcomes were compared between the two groups, and multivariable analyses were performed to elucidate the relevant factors for postoperative complications in several subgroups.

**Results:**

A total of 1172 patients were divided into those who underwent RG (*n* = 152) and those who underwent laparoscopic gastrectomy (LG) (*n* = 1020). Baseline characteristics were similar in the two groups, except the RG group included more patients undergoing total/proximal gastrectomy (TG/PG) and patients at clinical stage III. Compared with the LG group, the RG group had lower incidences of postoperative complications ≥ Clavien-Dindo grade III (2/152 (1.3%) versus 72/1020 (7.1%); *P* = 0.004), and intraabdominal complications ≥ grade II (6/152 (3.9%) versus 119/1020 (11.7%); *P* = 0.004). Multivariable analysis revealed that RG was a significant relevant factor for reducing overall postoperative complications (≥ grade III) (odds ratio (OR) 0.16, *P* = 0.013), and intraabdominal complications (≥ grade II) (OR 0.29, *P* = 0.002). Subgroup analyses demonstrated that this tendency was enhanced in patients undergoing TG/PG (OR 0.29, *P* = 0.021) or at clinical stage II/III (OR 0.10, *P* = 0.027).

**Conclusions:**

RG reduces the incidence of postoperative complications compared with conventional LG and this tendency may be enhanced in technically complicated procedures with demanding anastomosis or D2 lymphadenectomy. Patients requiring such procedures would most benefit from RG.

The surgical feasibility and oncological efficacy of laparoscopic gastrectomy (LG) for gastric cancer have been validated in several pivotal studies. Studies have shown that LG is not inferior to open surgery in treating early-stage gastric cancer [1, 2] and locally advanced gastric cancer [3–5]. Furthermore, the use of LG has spread extensively worldwide due to faster patient recovery and less complications compared with open surgery. Robotic surgery is regarded as an evolved type of laparoscopic surgery that overcomes limitations in forceps movement, with excellent dexterity and precision gained from the use of articulated surgical instruments. Robotic gastrectomy (RG) has recently been rapidly implemented universally. In Japan, RG for gastric cancer has been reimbursed by the public health insurance since April 2018 based on the results of a multicenter prospective Japanese study that revealed favorable surgical outcomes of RG [6]. Since the initiation of this insurance coverage, many Japanese hospitals have introduced RG as a substitute for conventional LG.

Many studies have compared the clinical outcomes of RG and LG. Most such studies have confirmed that RG has comparable surgical safety or feasibility to conventional LG, but have failed to show obvious benefits of RG over LG [7]. Moreover, almost all previous studies have reported higher costs and longer operation time as drawbacks of RG compared with conventional LG [8]; cost-effectiveness is currently a particularly controversial issue. For that reason, many surgeons currently want to know when to apply RG. There is a possibility that RG may have the potential to reduce postoperative local complications compared with LG [9, 10]. In particular, the wristed surgical instruments combined with the tremor-reducing function in RG enables precise dissection near the pancreas and precise intracorporeal anastomosis, which may reduce postoperative intraabdominal complications. Moreover, the three-dimensional camera system in RG makes it easy to find the optimal dissection plane. A reduction in the morbidity rate may reduce the total medical expenses. However, even if RG enables more precise dissection and reduces the morbidity rate compared with LG, the current drawbacks of RG make it irrational to completely replace conventional LG with RG at present. Thus, there is a need to determine which patients are the best candidates for RG and would most benefit from its advantages. Few studies have evaluated the superiority of RG over LG from this viewpoint.

RG was first introduced at our hospital in 2014, and has been performed in more than 150 patients thus far. Consistent with our learning curve, RG has been applied to a broad spectrum of procedures, including total or proximal gastrectomy (TG/PG), surgery for advanced stage gastric cancer, or after preoperative chemotherapy. The present study aimed to identify the advantages of RG over LG, focusing on postoperative morbidity, to identify the optimal candidates for RG who would most benefit from RG.

## Materials and methods

### Study design and patients

The present study was a retrospective, single-institutional, case-controlled study. Consecutive patients with primary gastric cancer undergoing either robotic or laparoscopic radical gastrectomy at our department from June 2014 to June 2020 were enrolled, and their clinical and surgical data were retrieved from our prospectively collected in-house database. Patients who received concomitant pancreatectomy or had remnant gastric cancer were excluded. The patient cohort was divided into the RG group and the LG group for comparison. All surgeries were performed or supervised by experienced gastric surgeons certified by the Japan Society for Endoscopic Surgery [11]. LGs were performed not only by staff surgeons, but also by resident surgeons. However, TG/PGs were performed principally by staff surgeons due to technical intricacy. RGs were performed only by staff surgeons, as industry-authorized certification is required to perform robotic surgery. All LGs were performed under the public health insurance system. The first 20 cases of RG were financed by our hospital as an in-house phase II study (from June 2014 to January 2015), with eligibility restricted to clinical stage I disease. The next 41 cases of RG were performed under the advanced medical service system partially financed by the public health insurance as a multicenter phase II study in Japan (from October 2015 to December 2016) [6], with eligibility expanded to clinical stage I/II disease. Thereafter, all RG cases were completely under the public health insurance system (from April 2018 to June 2020), and the indication for RG was extended to clinical stage III disease, even after neoadjuvant chemotherapy; during this period, the choice of RG or LG was based on the patient’s preference or consent. Disease staging followed the TNM classification (eighth edition) [12]. The extent of lymph node dissection and station numbering followed the Japanese classification and guidelines [13, 14]. The present study was approved by the institutional review board of the National Cancer Center, and all patients provided comprehensively informed consent.

### Technical points of the surgical procedure: laparoscopic gastrectomy

The energy device was mainly ultrasonic scissors, occasionally combined with bipolar vessel sealing devices in patients with visceral obesity. Intestinal anastomoses were intracorporeally performed by linear stapler, using the overlap method for esophagojejunostomy or gastrojejunostomy in Roux-en-Y reconstruction, and the delta-shaped anastomosis for gastroduodenostomy. The reconstruction method after PG was chosen depending on the size of the remnant stomach or the length of the remaining esophagus; esophagogastrostomy with an anti-reflux procedure (double-flap method) was selected when both remnants were large enough, otherwise double-tract reconstruction was selected. In accordance with the Japanese gastric cancer treatment guidelines [14], D1+ lymphadenectomy was performed for patients with cT1N0M0, otherwise D2 dissection was performed.

### Technical points of the surgical procedure: robotic gastrectomy

**Fig. 1:**
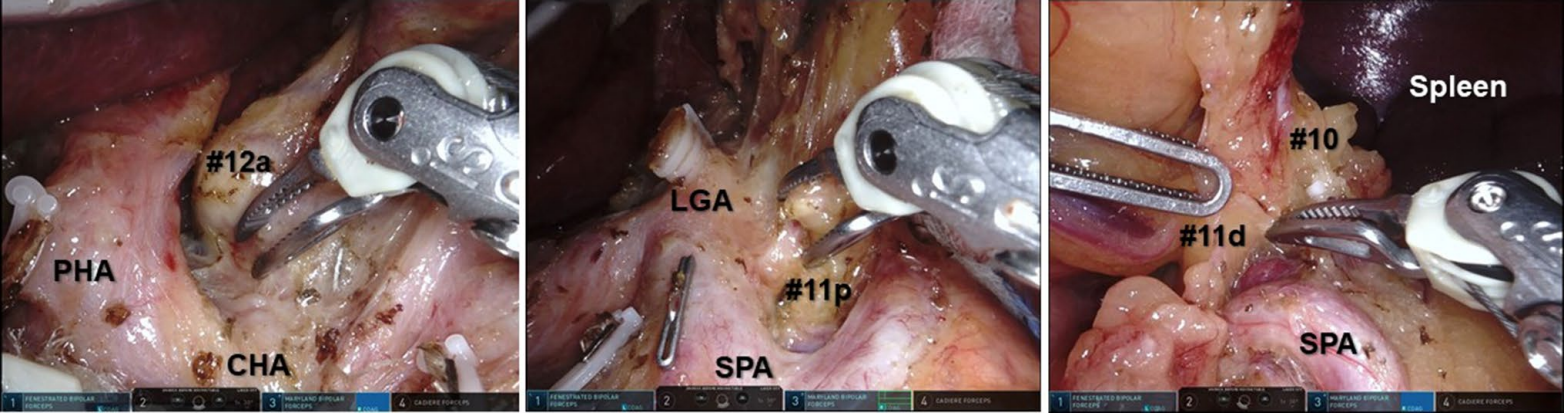
Double-bipolar technique for robotic lymph node dissection in the suprapancreatic region. The articulation of Maryland bipolar forceps is effectively used and activated with low-voltage mode. *PHA* proper hepatic artery; *CHA* common hepatic artery; *LGA* left gastric artery; *SPA* splenic artery

The DaVinci Si system was used in the initial 90 cases, and then the Xi system (Intuitive Surgical, Sunnyvale, CA, USA) was used. Regarding the energy device, the double bipolar method was fundamentally applied (Fig. 1), in which tissues were dissected by activated Maryland bipolar forceps as reported previously [15]. However, in contrast to the original method, the low-voltage coagulation mode was used for activation (i.e., soft coagulation). The reconstruction method basically followed the same procedures as in conventional LG. For the first 60 cases, conventional laparoscopic linear staplers were utilized for all anastomoses; subsequently, a specific robotic stapler (45 mm EndoWrist stapler; Intuitive Surgical) was used for esophagojejunostomy or gastroduodenostomy. The selection of the lymphadenectomy extent was the same as for LG.

### Evaluated outcomes

The primary outcome was postoperative complications within 30 days after surgery that were classified in accordance with the Clavien-Dindo (C-D) grading system [16]. The secondary outcomes were operation time, blood loss, drainage amylase levels on the 1st and 3rd postoperative days, length of postoperative hospital stay, conversion to open surgery, and number of harvested lymph nodes. Patients’ baseline, clinical, surgical, and pathological data were collected from the established database. These parameters were compared between the RG and LG groups.

### Multivariable analysis of the relevant factors for postoperative complications

Because of the heterogenity of the two groups, multivariable analysis was carried out to identify the relevant factors for postoperative complications (with the outcomes set as overall postoperative complications ≥ C-D grade III or postoperative intraabdominal complications ≥ C-D grade II). Univariate analyses were performed to identify the potential related factors, which were subsequently entered in the multivariable analysis. Such analyses were first conducted in the entire cohort, and then in the subgroup cohorts; i.e. patients undergoing TG/PG, with clinical stage II/III disease, undergoing distal gastrectomy (DG) for clinical stage I disease, with a high body mass index, or receiving preoperative chemotherapy to distinguish in which subgroups benefits to patients from RG stood out.

### Statistics

The chi-squared test or Student’s *t* test was used to compare values. Multivariable analyses were performed using the logistic regression model. All statistical analyses were performed using the JMP software program, version 14 (SAS Institute, Cary, NC, USA). All *P* values were two-sided, and *P* < 0.05 was considered statistically significant.

## Results

### Baseline data

Gastrectomy was performed in 1172 eligible patients during the study period, and this cohort was divided into the RG group (*n* = 152) and the LG group (*n *= 1020).

The baseline data are summarized in Table 1. The general background characteristics including sex and body mass index did not significantly differ between the two groups, and the median age was only 1 year younger in the RG group than the LG group (69 vs 70 years, *P* = 0.041). The stage distribution did not significantly differ between groups. Both groups comprised around 70% of patients with clinical stage I disease and 30% with clinical stage II/III disease, but the RG group tended to include more patients with clinical stage III disease (T3N(+) or T4aN(+)). Regarding the type of resection, the LG group included a higher proportion of patients undergoing DG, while the RG group included a higher proportion of patients undergoing TG/PG. In the RG group all of the surgeries were performed by staff surgeons, whereas in the LG group 38% were performed by resident surgeons as first operators. The RG group comprised more patients receiving preoperative chemotherapy.

**Table 1:** Baseline patient data

	Robotic (*n* = 152)	Laparoscopic (*n* = 1020)	*P*-value
Sex ratio (male:female)	95:57	707:313	0.087
Age (year)	69 [30–86]*	70 [23–91]*	0.041
Body mass index (kg/m^2^)	22.2 [17.0–30.7]*	22.7 [13.7–36.5]*	0.097
Clinical T factors			
T 1/2/3/4	87/27/30/8	609/176/178/57	0.969
Clinical *N* factors			
*N* (+)	29 (19.1%)	179 (17.5%)	0.619
Clinical stage			
I	109 (71.7%)	724 (71.0%)	0.635
II	19 (12.5%)	179 (17.5%)	
III	22 (14.5%)	108 (10.6%)	
IV	2 (1.3%)	9 (0.9%)	
Estimated tumor size (mm)	35 [10–120]*	35 [3–150]*	0.099
Type of resection			
Distal gastrectomy	87 (57.2%)	729 (71.5%)	0.001
Proximal gastrectomy	23 (15.1%)	112 (11.0%)	
Total gastrectomy	40 (26.3%)	154 (15.1%)	
Pylorus-preserving gastrectomy	2 (1.3%)	25 (2.5%)	
Operator			
Staff/resident surgeon	152/0	637/383	0.001
Preoperative chemotherapy	22 (14.5%)	60 (5.9%)	0.001

Data are presented as median [range]* or number (%)

### Surgical outcome and details of postoperative complications

Surgical outcome data are shown in Table 2. The RG group had a significantly longer operation time than the LG group. The amount of estimated blood loss did not differ between groups. The overall retrieved number of lymph nodes was higher in the RG group than in the LG group, and the number of lymph nodes harvested in PG and TG significantly differed between the RG and LG groups. The drain amylase levels tended to be lower in the RG group than the LG group, but this difference was not significant. The median length of postoperative hospital stay was the same in the two groups. In-hospital mortality occurred in one patient (0.1%) due to acute pulmonary embolism in the LG group, but none in the RG group. The RG group had significantly lower incidences of morbidities ≥ C-D grade III (1.3% vs 7.1%, *P* = 0.004) and ≥ C-D grade II (9.2% vs 19.5%, *P* = 0.004) than the LG group. In particular, intraabdominal complications (such as anastomotic leakage, intraabdominal abscess, and pancreatic fistula) were infrequent in the RG group. One reoperation was required in the RG group for incisional hernia at the port site on the 8th postoperative day.

**Table 2:** Surgical outcomes and morbidity

	Robotic (*n* = 152)		Laparoscopic (*n* = 1020)		*P*-value
Operation time (min)	341 [225–522]*		248 [136–722]*		< 0.001
Blood loss (g)	22 [3–176]*		15 [0–505]*		0.784
Conversion to open surgery, *n* (%)	0 (0)		8 (0.8%)		0.606
No. of harvested LNs					
Distal gastrectomy	35 [13–73]*		32 [6–115]*		0.258
Proximal gastrectomy	34 [11–54]*		26 [5–56]*		0.028
Total gastrectomy	52 [26–92]*		45 [16–104]*		0.025
Pylorus-preserving gastrectomy	36 [27–46]*		28 [12–50]*		0.188
Drain-amylase (IU/L)					
1 POD	334 [IQR 186—681]*		445 [IQR 223—895]*		0.196
3 POD	121 [IQR 58—225]*		131 [IQR 71—253]*		0.532
Postoperative hospital stay (days)	8 [6–46]*		8 [5–116]*		0.726
Pathological stage					
I/II/III/IV/complete response	103/32/15/0/2 (67.7/21.1/9.9/0/1.3%)		712/165/121/9/13 (69.8/16.2/11.8/0.9/1.3%)		0.574
In-hospital mortality, *n* (%)	0 (0)		1 (0.1)		0.699

Morbidity, *n* (%)	≥ CD III	≥ CD II	≥ CD III	≥ CD II	
Overall	2 (1.3)	14 (9.2%)	72 (7.1)	199 (19.5%)	0.004**
Anastomotic leakage	1 (0.7)	1 (0.7)	30 (2.9)	35 (3.4)	0.114**
Anastomotic stenosis	0 (0)	0 (0)	5 (0.5)	5 (0.5)	1.000**
Anastomotic bleeding	0 (0)	0 (0)	7 (0.7)	10 (1.0)	0.377**
Intra-abdominal bleeding	1 (0.7)	1 (0.7)	4 (0.4)	8 (0.8)	1.000**
Intra-abdominal abscess	1 (0.7)	3 (2.0)	24 (2.4)	53 (5.2)	0.101**
Pancreatic fistula	1 (0.7)	2 (1.3)	12 (1.2)	16 (1.6)	1.000**
Stasis	0 (0)	0 (0)	1 (0.1)	10 (1.0)	0.377**
Bowel obstruction	1 (0.7)	3 (2.0)	6 (0.6)	10 (1.0)	0.232**
Chylous ascites	0 (0)	0 (0)	2 (0.2)	5 (0.5)	1.000**
Cholecystitis	0 (0)	0 (0)	2 (0.2)	8 (0.8)	0.606**
Pneumonia	0 (0)	2 (1.3)	2 (0.2)	57 (5.6)	0.026**
Pleural effusion	0 (0)	0 (0)	3 (0.3)	8 (0.8)	0.606**
Thrombosis	0 (0)	0 (0)	2 (0.2)	4 (0.4)	1.000**
Wound infection	0 (0)	1 (0.7)	0 (0)	2 (0.2)	0.341**
Wound dehiscence	0 (0)	0 (0)	1 (0.1)	2 (0.2)	1.000**

Data are presented as median [range]* or number (%)

*LN* lymph node, *CD* Clavien-Dindo, *POD* postoperative day, *IQR* interquartile range

**P value for the comparison of morbidities classified as ≥ Clavien-Dindo grade II

### Relevant factors for postoperative complications

**Fig. 2:**
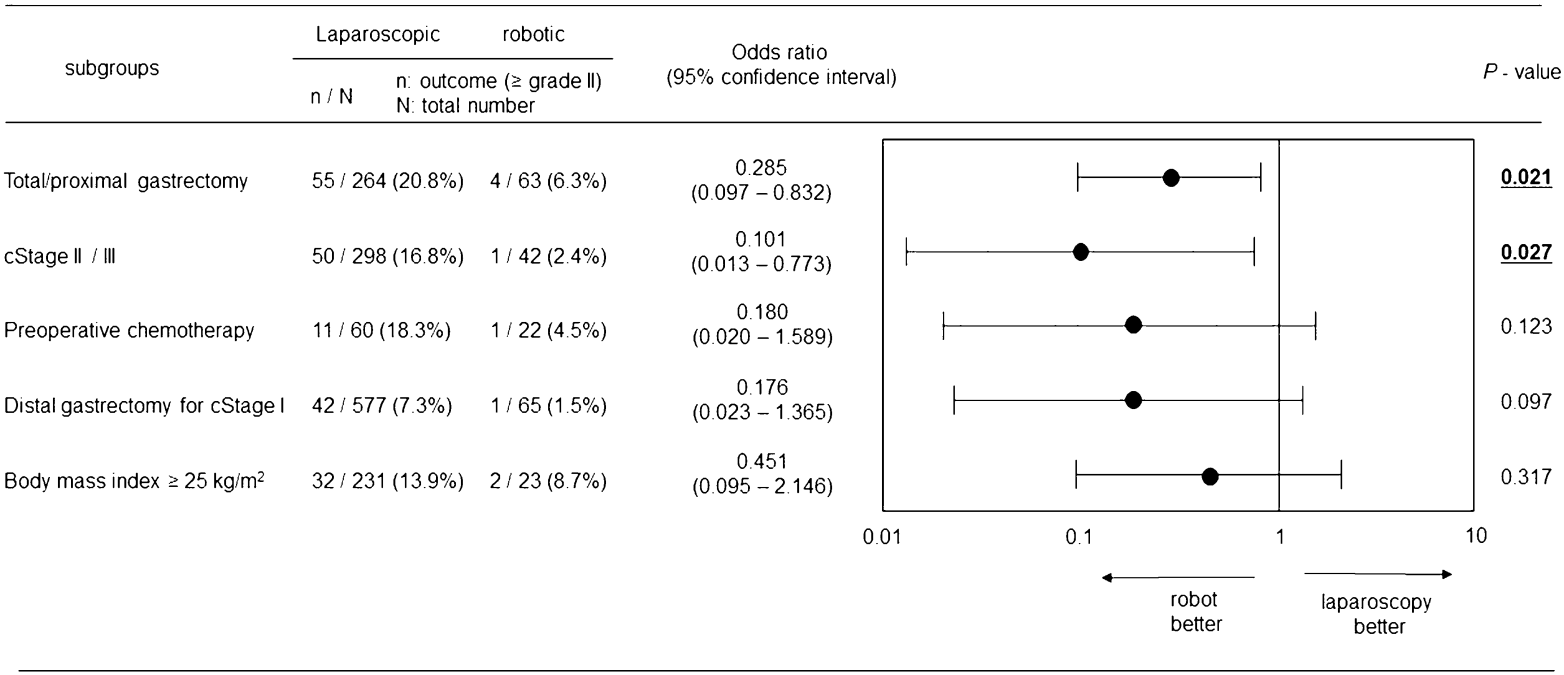
Odds ratios of the use of robotic surgery to cause postoperative intraabdominal complications ≥ Clavien-Dindo grade II in subgroups

The results of relevant factors analyses for overall postoperative complications ≥ C-D grade III in the entire cohort (*n* = 1172) are summarized in Table 3. Multivariable analysis revealed that the significant relevant factors for postoperative complications were male sex (odds ratio (OR) 3.15), TG/PG (OR 2.37), clinical stage II/III disease (OR 1.82), and robotic surgery (OR 0.16). As our clinical experiences showed the usefulness of RG in TG/PG, the same analysis was performed in the cohort limited to patients undergoing TG/PG (*n* = 327) (Table 4); similarly, male sex (OR 3.82), clinical stage II/III disease (OR 2.13), and robotic surgery (OR 0.12) were identified as significant relevant factors for postoperative complications in this subgroup. An analysis of the relevant factors for intraabdominal complications ≥ C-D grade II was conducted in the entire cohort (*n* = 1172), as this type of complication may directly reflect the influences of surgical manipulation in the abdominal cavity; again, male sex (OR 2.01), TG/PG (OR 2.33), and robotic surgery (OR 0.29) were identified as significant relevant factors (Table 5). Throughout these analyses, “use of robotic surgery” was consistently identified as the factor associated with reduced complications. Therefore, the OR of robotic surgery to laparoscopic surgery for causing intraabdominal complications ≥ C-D grade II was examined in subgroup cohorts of patients undergoing TG/PG, receiving preoperative chemotherapy, undergoing DG for clinical stage I disease, with clinical stage II/III disease, and with a high body mass index in order to examine in which subgroups this tendency was considerable. In all of the subgroup cohorts, the OR detected by multivariable analyses was declined (Fig. 2). Among them a statistical significance (the upper limit of the 95% confidence interval (CI) < 1.0) was detected in the TG/PG subgroup (OR 0.285, 95% CI 0.097–0.832) and clinical stage II/III subgroup (OR 0.101, 95% CI 0.013–0.773). In contrast, such a statistical result was not observed (OR of 95% CI range crossing 1.0) in the other subgroups.

**Table 3:** Univariate and multivariable analyses of the relevant factors for overall postoperative complications (≥ Clavien-Dindo grade III) in the entire cohort (*n* = 1172)

Risk factors	Number	Univariate analysis		Multivariable analysis	
		Odds ratio (95% CI)	*P*-value	Odds ratio (95% CI)	*P*-value
Sex (male)	800	3.55 (1.75–7.20)	< 0.001	3.15 (1.54–6.43)	0.002
Age (≥ 75 year-old)	317	0.99 (0.59–1.70)	0.997		
Body mass index (≥ 25 kg/m^2^)	254	1.58 (0.94–2.65)	0.084		
Resection extent (total/proximal gastrectomy)	327	2.63 (1.63–4.23)	< 0.001	2.37 (1.45–3.91)	< 0.001
Preoperative chemotherapy (presence)	82	1.43 (0.63–3.21)	0.393		
Clinical stage (stage II / III)	340	2.2 (1.37–3.54)	0.001	1.82 (1.11–2.99)	0.018
Surgical procedure (robotic)	152	0.17 (0.04–0.72)	0.016	0.16 (0.04–0.68)	0.013
Operator (resident surgeon)	414	0.61 (0.35–1.05)	0.075		

*CI* confidence interval

**Table 4:** Univariate and multivariable analyses of the relevant factors for overall postoperative complications (≥ Clavien-Dindo grade III) in patients undergoing total/proximal gastrectomy (*n* = 327)

Risk factors	Number	Univariate analysis		Multivariable analysis	
		Odds ratio (95% CI)	*P*-value	Odds ratio (95% CI)	*P*-value
Sex (male)	245	4.12 (1.23–13.82)	0.022	3.82 (1.12–13.07)	0.033
Age (≥ 75 year-old)	82	1.36 (0.64–2.91)	0.423		
Body mass index (≥ 25 kg/m^2^)	61	0.86 (0.34–2.16)	0.746		
Preoperative chemotherapy (presence)	38	1.62 (0.63–4.19)	0.321		
Esophageal invasion (presence)	48	1.79 (0.76–4.21)	0.180		
Clinical stage (stage II/III)	137	2.41 (1.18–4.89)	0.015	2.13 (0.98–4.64)	0.057
Surgical procedure (robotic)	63	0.11 (0.01–0.79)	0.028	0.12 (0.02–0.92)	0.042

*CI* confidence interval

**Table 5:** Univariate and multivariable analyses of the relevant factors for postoperative intraabdominal complications (≥ Clavien-Dindo grade II) in the entire cohort (*n* = 1172)

Risk factors	Number	Univariate analysis		Multivariable analysis	
		Odds ratio (95% CI)	*P*-value	Odds ratio (95% CI)	*P*-value
Sex (male)	800	2.31 (1.43–3.73)	< 0.001	2.01 (1.23–3.27)	0.005
Age (≥ 75 year-old)	317	1.02 (0.67–1.55)	0.922		
Body mass index (≥ 25 kg/m^2^)	254	1.42 (0.93–2.17)	0.102		
Resection extent (total/proximal gastrectomy)	327	2.64 (1.81–3.86)	< 0.001	2.33 (1.52–3.58)	< 0.001
Preoperative chemotherapy (presence)	82	1.49 (0.79–2.85)	0.219		
Clinical stage (stage II/III)	340	1.83 (1.25–2.68)	0.002	1.39 (0.89–2.14)	0.141
Surgical procedure (robotic)	152	0.36 (0.16–0.78)	0.009	0.29 (0.13–0.64)	0.002
Operator (resident surgeon)	414	0.53 (0.35–0.81)	0.004	0.71 (0.43–1.18)	0.185

*CI* confidence interval

## Discussion

The present study examined the surgical outcomes of minimally invasive surgery (RG and LG) in a relatively large patient cohort. Postoperative complications were markedly less frequent in the RG group than the LG group, even though the RG group included more technically complicated cases, such as patients undergoing TG, those with clinical stage III disease, and those who had undergone preoperative chemotherapy. Multivariable analyses revealed that the use of RG was strongly associated with the reduced occurrence of postoperative complications. Furthermore, this tendency was more obvious in the TG/PG and clinical stage II/III subgroups. Although the present study was retrospective, the present results indicate that a reduction in postoperative complications is a vital advantage of RG over conventional LG, and that this advantage is enhanced in technically complicated procedures with demanding digestive anastomoses or D2 lymphadenectomy. Probably, the difference between RG and LG is less likely to appear in technically simple procedure (e.g. DG for early-stage disease), because the incidences of postoperative complication are low enough even in conventional surgery. Alternatively, the difference is likely to be recognizable in technically difficult procedures which are potentially with high incidences of postoperative complications in conventional surgery.

Longer operation time and higher costs are negative aspects of RG. The median operation time was longer by about 100 min in the RG group than the LG group in the current study. In accordance with our learning curve, the difference in operation time between the two groups became smaller over time, but did not reach a completely equivalent level. The reason for this issue may be that the extended operation time in RG derived from so-called junk time, comprising the time required to set the systems, reposition the surgical arms, change instruments, or deal with instrument problems [17]. Such delays will likely be reduced when more sophisticated or simpler robotic surgical systems are launched. Meanwhile, in the current study, the exact costs were not assessed for comparison. A multicenter prospective study in Japan reported that the total medical cost for RG is 1,799,628 JPY [6], and in other countries, it is estimated that the medical costs for RG are about twice as high as for conventional LG [18–21]. In order to solve this problem, efforts to lower prices by the industries are expected.

Considering these current disadvantages of RG, it is essential to evaluate whether RG is superior to conventional procedures. Numerous publications, including retrospective studies and meta-analyses, have concluded that RG is feasible and safe, with surgical outcomes almost equal to traditional LG [7, 8, 22–24]. Some studies reported a larger number of harvested lymph nodes in the RG group than the LG group [6, 25], which may be one of the advantages of RG suggesting accuracy of lymphadenectomy; the current study also showed the same tendency, particularly in TG or PG. When treating cancer in the upper stomach, the operative fields (such as the splenic hilum and esophageal hiatus) are deeply located, which may restrict the use of conventional straight laparoscopic instruments. Articulating robotic devices may facilitate more precise maneuvers, resulting in accurate lymph node dissection. However, the oncological effectiveness of RG should be evaluated through long-term follow-up.

Most previous studies and meta-analyses have concluded that the incidences of postoperative complications are equal between RG and conventional LG [7, 8, 22–24]. However, similarly to the current study, some Japanese studies have suggested that postoperative complications are reduced in RG compared with LG [6, 10]. We consider reduction of postoperative complication as the most valuable benefit of RG. A Japanese multicenter prospective trial that included 326 patients with stage I/II gastric cancer reported an overall rate of morbidities ≥ grade III of 2.45% in RG, which is significantly less than historical data of LG (6.4%) in the same population [6]. Furthermore, one research group in Japan reported a significantly lower incidence of postoperative complications in RG (2.3%) compared with LG (11.4%) [10]. They additionally reported RG is reportedly more effective in reducing postoperative complications in TG than in DG [10], which supports the results of the current study.

One possible explanation for this consistency may be the choice of the dissecting energy device. The double bipolar technique was used in both the present study and the study by the other research group [15]. In contrast to ultrasonic devices, which are probably used at most other institutions, bipolar forceps are able to effectively use a wrist function. The use of this wristed bipolar device might assist in optimal dissection even with D2 extent for advanced cancer while being gentle to the preserved organs, such as the pancreas. According to our experiences, the shaft of the completely straight harmonic device in RG is likely to press on the pancreas, presumably causing mechanical damage to the parenchyma, which may result in the same situation as in conventional LG. We speculate the use of double bipolar technique was associated with encouraging outcomes in the stage II/III subgroup in the current study. However, even if robotic system may enable D2 lymphadenectomy for advanced cancer, it should be noted that the current robotic instruments lack haptic feedback to surgeons. We suppose that large tumors with serosal infiltration are not appropriate indication for RG due to difficulty of tumor handling as well as potential risk of cancer cell spillage.

In the current study, favorable outcomes were obtained with no anastomotic leakage in robotic TG/PG, which generally requires technically demanding anastomoses. In most intracorporeal anastomoses, the entry hole was sutured after side-to-side linear stapling. The use of an articulated needle driver in RG may have enabled more precise and accurate suturing closure. TG is known to have higher incidences of postoperative complications (including leakage) than DG. If RG is actually able to decrease the incidences of such complications, this will be of great significance in clinical practice.

The present study has several limitations. First, there was slight heterogeneity in the backgrounds of the two groups, and potential selection bias could not be excluded. Therefore, the results should be interpreted cautiously. However, the RG group included a greater number of technically difficult procedures than the LG group, which suggests that the effectiveness of RG was not overestimated. Meanwhile, RG was entirely operated by staff surgeons, but around 40% of LG was by resident surgeons. This fact might influence the surgical quality. To control these confounding factors, multivariable analyses were carried out in which factors associated with “technical difficulty” as well as “operator” were incorporated as variables. Consequently, RG was throughout detected as a significant relevant factor to reduced postoperative complication; even in the TG/PG subgroup which operators were limited to staff surgeons. Propensity score matching was not employed because of the small number of important events in the RG cohort. Second, this was a single-center retrospective study in a Japanese high-volume center. The volume of gastric cancer surgery differs between Eastern and Western countries and between institutions; hence, the current results may not be straightforwardly generalizable to other patient populations. Future progress of auto intelligence technologies may help to bridge this gap. Third, long-term oncological outcomes were not evaluated. Although previous studies have reported comparable long-term outcomes between RG and LG [26, 27], such studies are still scarce. As postoperative complications are related with unfavorable prognoses [28], further evaluation of the long-term outcomes of RG is required. Therefore, we aim to closely follow the present cohort, and to perform a prospective larger-scale study in a multicenter setting in the future. The Japan Clinical Oncology group is currently running a randomized trial to evaluate whether RG is superior to LG (JCOG1907: UMIN000039825), with eligibility up to the T2N2 stage, which will provide very useful information; however, another prospective study including a considerable number of stage II/III patients is warranted.

In conclusion, a reduction in postoperative complications may be an important advantage of robotic surgery over conventional laparoscopic surgery; this tendency may be enhanced in technically complicated procedures with demanding anastomosis or D2 lymphadenectomy. Considering all, patients requiring such procedures would benefit from RG and seem to be adequate candidate for RG. Of course, the indication should be extended step by step in each institution with understanding distinct features of robotic devices for patient’s safety.
